# Nitrous-Oxid-Oxygen Anesthesia by the Method of Rebreathing with Special Reference to the Prevention of Surgical Shock

**Published:** 1910-04-15

**Authors:** Willis D. Gatch

**Affiliations:** Baltimore


### NITROUS-OXID-OXYGEN ANESTHESIA BY THE METHOD OF REBREATHING WITH SPECIAL REFERENCE TO THE PREVENTION OF SURGICAL SHOCK.

WILLIS D. GATCH, M.D., BALTIMORE.

The following article gives the results of a trial of nitrous-oxid -oxygen anesthesia in the service of Professor Halstead at the Johns Hopkins Hospital. My object at the outset was to develop a method of administering these gases so simple, cheap and effective as to make possible their more general use. As nitrous-oxid and oxygen are expensive and are to be obtained only in heavy cylinders, it seemed to me that the solution of the question of their administration, so far as cost and convenience are concerned, must lie in using them over instead of wasting them after one inhalation. I have, therefore, studied the effects of rebreathing these gases with a view to determining to what extent this may be permitted without injury to the patient. My results have led me to believe that within certain limits the method is not only harmless but beneficial.

For the sake of clearness I shall consider the facts to be presented under three headings, namely: (1) the apparatus employed; (2) the method of administration; (3) the clinical results.

### THE APPARATUS

This consists of a holder for the cylinders of gas, a rubber bag, a face piece, and the connecting tubing. The holder, consists of a basket of iron, triangular on cross-section, into which two cylinders of nitrous oxid and one of oxygen may be placed. Small set screws hold them in position. One of the lateral bars of the basket is made into a handle for carrying it about. To the upper part of the holder is bolted an L-shaped tube, each arm of which is about 2 inches long and 1 inch in diameter. Three small tubes through which gas is admitted from the cylinders enter the upper part of the L tube. Each of these is connected with its cylinder by means of a short piece of rubber tubing and an ordinary collar of the kind sold with the cylinder.

A rubber bag of 6 to 8 liters' capacity is attached to the vertical arm of the L tube, and a rubber pipe one inch in diameter and 2½ feet long to the horizontal arm. This pipe is wrapped with wire to prevent kinking. It leads to the face piece. If so desired, the rubber bag may be attached directly to the face-piece. It is more convenient, however, to have it on the holder. There is no obstruction to breathing through a tube an inch in diameter. As the face piece is the most important part of the apparatus, it merits a somewhat careful description.

It consists of a valve box and a mask. The valve box is made on the principle of a trombone and has an outer and inner tube. By sliding the latter to a given one of three positions, the patient may be made to breathe air in and out through valves; or gas (with oxygen if desired) in and out through valves, or gas to and fro into the bag.

The inner tube may be pushed into three different positions: When in the first position, air enters the valve box through the air vent, and is breathed inward through the valve V, and outward through the valve and the holes in the diaphram. Gas is usually not given when the air vent is open. In the second position, the air-vent is closed and gas is breathed inward and outward just as air is breathed the first position, the only difference being that the orifice is over the opposite end of the large opening, in the outer tube. In the third position, the air vent is still closed and the valves are thrown out of action. Gas is then breathed to and fro into the bag.

The mask is attached directly to the valve-box. It is essential that this fit the patient's face so as to be absolutely air-tight. The ordinary face-piece is fitted with an inflatable rubber rim. As such a mask cannot possibly fit every face, a set of graduated sizes must be employed. Even then a mask cannot always be found which will fit the face of an edentulous patient or of one with a beard or a prominent nose. As these face-pieces do not fit under the chin, they are usually brushed away by any sudden movement of an unruly patient. Furthermore ,it is very tiresome to hold such a mask accurately in place during a long anesthesia.

I have hit on a simple device which does away with these difficulties. This consists of a mask provided with a rubber cuff, and big enough to fit the largest face. The cuff is pulled up over the margin of the mask. To adjust the cuff to the face the free portion of the cuff is turned back over the margin of the mask, and the latter is put on the face so as to take in the nose and chin. The cuff is then turned down, when it firmly grasps the chin, cheeks and nose. It may be made to fit any face. I have used it on a child 2 years old. The cuff allows dental props, gags, etc., to be put into the mouth without admitting air.

One other part of the mask—the ether attachment—remains to be described. This consists of a cylinder of wire gauze an inch and a half in diameter, fitted at one end to a short metallic cartridge. The latter fits accurately the inside of a tube passing through the wall of the mask. Tube and cartridge are perforated by small openings which may be super-imposing by rotating the cartridge. The wire cylinder is packed with gauze, on which ether is dropped through the orifice H. The latter is provided with a funnel, inside of which is a perforated plate which prevents any splashing of ether when the patient expires. Because of the heat inside the mask, the ether is rapidly evaporated from the gauze, and very small amounts are required. It need hardly be said that chloroform should never be given with such an apparatus.

The main features of this apparatus may be summarized as follows:

1. It is simple and is made to withstand hard usage. It cannot easily get out of order, and, if it does, it may be quickly taken apart and repaired.

2. It is light and easily portable. Exclusive of the cylinder, it weighs only six pounds; with two cylinders, 26 pounds. Packed in a bag of canvas or leather it may be taken anywhere.

3. It may be quickly sterilized by boiling. To spare the rubber we usually sterilize the bag and tubing with a solution of bichloride of mercury which is rinsed away with water.

4. It is economical in the use of gases, as the cuff prevents all leakage.

5. There is so little mechanism to be considered and the administration is so simple that the anesthetizer has plenty of time for observation of his patient.

6. It is inexpensive. The total cost of the apparatus is about $25.

With the air vent open the cuff of the mask is fitted to the patient's face, care being taken to prevent the admission of air at the sides of the nose. In some cases it may be necessary to lay a piece of gauze across the bridge of the nose and draw the cuff over it, or to hold the cuff there with the finger. The inner tube of the valve-box is pushed to its mid-position and nitrous oxid admitted to the bag. The patient now inhales this gas and expires into the outer air, thus washing out, as it were, all the air from his lungs. This process is continued until he becomes very slightly cyanotic. Then the inner tube is pushed to its final position and the patient breathes to and fro into the bag. At this moment a small puff of oxygen is admitted to the bag, just enough to restore the natural color of the face. The patient now rebreathes a mixture of nitrous oxid and oxygen until the inner tube of the valve box is moved back to its mid-position. He then exhales each breath into the air until the bag is empty. The anesthetizer then fills it with a fresh mixture of gases, which the patient again rebreathes. No attempt is made to measure the exact percentage of oxygen given. This we regard as unnecessary. It is perfectly easy to add directly from the oxygen cylinder exactly the right amount of this gas to each bag of nitrous oxid. The patient's color is an extremely delicate indicator of the amount of oxygen he is getting. Our rule is to give just enough oxygen to keep the patient's color free of the least tint of cyanosis. The most elaborate device for regulating the percentages of the two gases can do nothing more than this.

The question now arises whether rebreathing in the manner I have just described is injurious. Many writers have attached great importance to certain volatile organic poisons in the expired air. This has caused several inventors of apparatus for giving nitrous oxid and oxygen to do away entirely with rebreathing. Hewitt's apparatus, although its inventor states that rebreathing is probably not harmful, does not permit rebreathing. Dr. H. Warren Buckler, anesthetist for Dr. H. A. Kelly, has used the method of rebreathing for several years and has noted no ill effects therefrom. I have sought from the first to determine to what extent rebreathing is permissible. Prof. W. H. Howell has reviewed the experimental data bearing on the question of the harmful products of expired air. His conclusion, which is based on the experimental work of Haldane and Smith, is that it is highly improbable that any volatile organic poisons exist in the breath, and that the only harmful products of respiration are water vapor and carbon dioxid. He says:

Individuals, kept in a confined space for a number of hours, give no symptoms of evil effects except when the accumulation of carbon dioxid has reached a concentration of over 4 per cent. At this concentration rapid breathing is apparent, and if the carbon dioxid rises to 10 per cent. great distress is felt, and the face becomes congested and blue.

If we accept these conclusions as correct, we may make a rough calculation based on them to determine the number of times a patient may rebreathe a given quantity of gas without injury. The average volume of an expiration for an adult is 500 c.c. This contains 4 per cent., or 20 c.c., of carbon dioxid. Therefore an adult patient can breathe the contents of a bag containing 8 liters of gas sixteen times before the carbon dioxid content of the same will reach 4 per cent.

At first I adhered pretty closely to this rule, as I feared that too high a percentage of carbon dioxid would be injurious. I also tested frequently, usually in the course of the same anesthesia, the effect of rebreathing and of a continuous administration without rebreathing. The latter procedure has always been the less satisfactory one. With it the patient's respiration soon becomes shallow and his pulse more rapid, even when his color is good and the anesthesia deep and satisfactory. With breathing on the other hand, the respiration becomes deep and full and the pulse rate usually falls. This result, until I had familiardize myself with the recent work of Dr. Yandell Henderson on "Shock", I attributed entirely to the stimulant action of carbon dioxid on the respiratory center. Henderson's work, however, indicates that this compound has also a direct action on the venous system.

"Failure of the circulation in shock," he states, "is due primarily to abolution of venous, not arterial, tonus." "The hypothesis is presented that acapnia (diminished carbon dioxid in the blood and tissues resulting from hyperpnea and from exhalation of carbon dioxid from exposed eviscera) is the cause of surgical shock." Carbon dioxid he believes, is the normal stimulant of the venous wall if the amount of this gas in the blood decreases below a certain level, the veins dilate and the blood accumulates in them until not enough blood is returned to the heart properly to support the circulation. Shock would thus only indirectly be the result of trauma. When it occurs in the course of anesthesia or after a painful injury it would be due to over-ventilation of the lungs, which diminishes the carbon dioxid content of the blood. Henderson was able, merely by regulating "the rate of pulmonary ventilation" to adjust the heart to any desired rate of beat. He be-believes that carbon dioxid exerts a stimulative action, not only on the respiratory center, but on the vasomotor and cardo-inhibitory centers as well. The respiratory center is, however, much more sensitive to the action of carbon dioxid than the others, and not until the departure from normal of the carbon dioxid content of the blood becomes fairly well marked is the pulse slowed or the blood-pressure elevated. Thus a reduction of the carbon dioxid content of the blood causes, as a rule, shallow, feeble respiration, a rapid pulse, and low blood-pressure, while excess of carbon dioxid causes deepened respiration, slowing of the pulse and increase of blood-pressure.

It is evident, if this theory be correct, that rebreathing to a certain extent, during nitrous-oxid-oxygen anesthesia, is not harmful, but beneficial. The respiratory rate with this form of narcosis is very rapid—frequently from forty to sixty a minute. Such overventilation of the lungs must rapidly take carbon dioxid from the blood. I shall briefly present certain observations which lend support to Henderson's theory.

1. Rebreathing, provided enough oxygen be given to prevent cyanosis, can be permitted for a surprisingly long time without any appreciably injurious result. I have tested this by having healthy adults breathe to and fro the contents of a six-liter bag of oxygen. This may be done from four to eight minutes without the slightest change in pulse or blood-pressure. The gas in the bag becomes hot, moist and disagreeable to breathe, and there is some sweating; otherwise there is no discomfort from this experiment.

2. The pulse, respiration and blood-pressure, once anesthesia is well established, are very little affected by the trauma of the operation. I have twice given this anesthetic during the evulsion of large peripheral nerves and neither time was there the least change in the patient's condition. Likewise there is no change during the most vigorous manipulations of ankylosed joints. Little effect is produced by trauma and exposure of the intestines. This would seem to be favorable to Henderson's contention that trauma produces shock chiefly by increasing the ventilation of the blood in the lungs. Rebreathing prevents such ventilation.

3. A pulse, rapid before operation, is nearly always slowed when rebreathing is permitted for some length of time. Very sick patients, with rapid pulse and quick, shallow respiration, actually seem benefitted by this form of anesthesia. A young man with gangrene of the bowel had a pulse-rate before operation of 160. During the operation, which lasted forty-five minutes, his pulse varied from 90 to 120 and was of a distinctly better quality than before. If the anesthesia is unsatisfactory, or if rebreathing is not permitted to any great extent, I have always noted an acceleration of the pulse. This observation is in accord with Hewitt's statement, that "the pulse is invariably accelerated." It is to be noted that Hewitt's apparatus does not permit rebreathing.

On two occasions I have observed a sudden and marked slowing of the pulse, in one case from 90 to 60 and in another from 80 to 50. In both cases the tension was also markedly increased. Both patients were of a good color and both had been rebreathing for rather long intervals. I attribute a fall of this kind to a sudden stimulation of the cardio-inhibitory center by an excess of carbon dioxid in the blood. On washing out the lungs with a fresh supply of gas, the pulse-rate immediately rose. The blood seems to free itself of an excess of carbon dioxid with great rapidity.

4. There is usually a well-marked and sustained rise of blood-pressure during this form of narcosis. In the case of the patient whose chart is shown in Figure 4 the rise was from 100 to 120. I have a number of charts which illustrate this fact. According to Henderson, carbon dioxid has a direct stimulant action on the vasomotor center.

The carbon dioxid is not the only product of expiration the effect of which must be studied with regard to rebreathing. The heat of the exhaled gases has also an important action. Normally the body loses a large amount of heat in the expired air. This loss is prevented by rebreathing. Therefore there is a rise of body temperature. This I have almost always found to be over half a degree in anesthesias of thirty minutes or more. During an administration of three hours the temperature rose 1.3 degrees.

Rebreathing is thus a most effective method of heating the gases, which are cold on being freed from their cylinders.

The body also loses considerable moisture by way of the lungs. Water vapor accumulates in the rebreathed gases, but this, so far as I have observed, has no ill effect on the patient.

It is best and cheapest not to admit any air during the administration. Satisfactory anesthesia is impossible with enough air to prevent cyanosis, because of the large amount of nitrogen in the air.

### CLINICAL RESULTS AND OBSERVATIONS

We have given nitrous oxid with oxygen by the method of rebreathing without mishap to about 700 patients. Records of the pulse, respiration and blood-pressure have been kept during the more important administrations, and postoperative examinations of the urine made. From the data so obtained I shall attempt to answer three questions: 1. Is this form of anesthesia safe and free from bad after-effects? 2. Is it deep and smooth enough to be satisfactory to the surgeon? 3. Is the administration sufficiently cheap and convenient to be generally used?

It would be foolish to assert that there is no danger. The danger lies, however, not at all in the anesthetic, but entirely in the way it is given. There are three danger signals: cyanosis, slowing of the pulse, and vomiting. With a good color, a pulse of 70 or above, and regular breathing, there is no danger. Vomiting is not a cause of trouble when the patient has been properly prepared for operation. It is to be feared if he has just eaten, or if he has intestinal obstruction. At the first sign of retching the mask is to be removed. As the patient regains consciousness very quickly, he is not liable to aspirate vomitus.

For an adult man, rebreathing can be permitted for two-minute intervals, and for three-minute intervals for women and children.

I have met practically no bad after-effects. A few patients have vomited during or just after the administration. A few have had slight headache. A boy with extensive burns was anesthetized daily for twenty to fifty minutes on three successive days without the slightest appreciable evil result. He could take food immediately after the administration. Although a very large percentage of our patients have been seriously ill at the time of the operation, many of them with advanced pulmonary or renal disease, we have had no fatalities of postoperative complications attributable to the anesthetic.

In answer to the second question I may state in a general way that for the cases for which this anesthetic is especially indicated it is as satisfactory to the surgeon as ether. Almost every abdominal and peripheral operation except craniotomy and operations on the female pelvic organs, has been performed under it. Fifty of the cases have been laparotomies. The list has included drainage of the mastoid cells, drainage of the antrum of Highmore, excision of tuberculous glands of the neck, drainage of empyema, removal of the breast for benign and malignant disease, operations on the gall-passages, intestinal anastomosis, appendectomy, cystotomy, nephrectomy, repair of typhoid perforation of bowel, cure of hernia, castration, amputations of leg and arm, exploration of great joints and evulsion of nerves. One administration lasted three hours; three others have been for more than two hours. Most failures are to be traced to a poorly fitting mask. A very small amount of air will spoil the anesthesia. It would be claiming too much, however, to assert that this form of narcosis is always as satisfactory as that of ether. Certain patients cannot be kept properly relaxed without a disagreeable degree of cyanosis. With such cases it is wisest not to go on very long with nitrous oxid, but to give ether at once, either very small amounts in the mask or by the drop method. Ether should never be given for a long time by means of the mask. By the aid of a few drops, however, there is rarely a patient who cannot be carried through the more difficult periods of the operation.

To the third question I can at once answer in the affirmative. For the following reasons this form of anesthesia is very convenient:

1. It is pleasant to the patient; many patients will consent to operation under "laughing gas" who would refuse if ether were to be used.

2. Consciousness is quickly lost and quickly regained. The patient does not have to be watched by the anesthetizer for more than a brief period after the operation, and may be allowed to come out of the anesthetic at any time should his cooperation be necessary to the surgeon. Very few patients have any excitement on recovery.

3. The anesthetic is so nearly harmless that one does not hesitate to give it to the same patient over and over again, at frequent intervals, or to use it for painful dressings, manipulations, etc.

4. The apparatus is cheap, easily portable, simple and not difficult to use.

5. The cost is not great. The patient consumed one cylinder of nitrous oxid (cost $1.75) and ten to fifteen gallons oxygen (cost about 40 cents) in two hours and fifteen minutes. Roughly, the cost is about 1½ cents per minute of anesthesia. The cost is greatly lessened by the method of rebreathing.

Besides being safe and convenient, this form of anesthesia has, I believe, a unique characteristic which makes it especially valuable for very sick patients. I refer to its remedial action for shock. This effect, which has already been discussed, may make of the anesthetic an aid instead of an evil. Furthermore, I have seen no cases of post-operative collapse following its use. Patients who cannot be easily anesthetized by nitrous oxid alone may first be etherized to a moderate depth and the anesthesia then continued with nitrous oxid. The therapeutic effect of rebreathing may thus be secured for all cases.

SUMMARY

1. Nitrous oxid with oxygen may be successfully given by a very simple and easily portable apparatus.

2. It is unnecessary to measure the percentage of oxygen given.

3. There are no harmful organic substances in expired air.

4. Rebreathing to a moderate degree is harmless.

5. In many cases rebreathing is beneficial, as it causes increased depth of respiration, slowing of the pulse, a rise of the blood-pressure and a rise of temperature. It seems probable that all but the last of these results may be attributable to the action of carbon dioxid.

6. Evidence is presented which indicates that surgical shock in the absence of hemmorrhage may be caused in part by excessive pulmonary ventilation.

7. The method has met the requirements of a thorough clinical test.

8. The ether-nitrous-oxid sequence is suggested for certain cases.

I desire to thank Professor Halsted and Prof. W. H. Howell for criticism of this article and Professor Halsted for the use of the clinical material on which it is based.—*Journal, A.M.A.* March 5th, 1910.

